# The Prevalence of Disease Clusters in Older Adults with Multiple Chronic Diseases – A Systematic Literature Review

**DOI:** 10.1371/journal.pone.0079641

**Published:** 2013-11-11

**Authors:** Judith Sinnige, Jozé Braspenning, François Schellevis, Irina Stirbu-Wagner, Gert Westert, Joke Korevaar

**Affiliations:** 1 NIVEL, Netherlands Institute for Health Services Research, Utrecht, the Netherlands; 2 IQ Healthcare, Scientific Institute for Quality of Healthcare, Radboud University medical centre, Nijmegen, The Netherlands; 3 Department of General Practice and Elderly care medicine/EMGO Institute for Health and Care Research, VU University Medical Center, Amsterdam, The Netherlands; Iran University of Medical Sciences, Islamic Republic Of Iran

## Abstract

**Background:**

Since most clinical guidelines address single diseases, treatment of patients with multimorbidity, the co-occurrence of multiple (chronic) diseases within one person, can become complicated. Information on highly prevalent combinations of diseases can set the agenda for guideline development on multimorbidity. With this systematic review we aim to describe the prevalence of disease combinations (i.e. disease clusters) in older patients with multimorbidity, as assessed in available studies. In addition, we intend to acquire information that can be supportive in the process of multimorbidity guideline development.

**Methods:**

We searched MEDLINE, Embase and the Cochrane Library for all types of studies published between January 2000 and September 2012. We included empirical studies focused on multimorbidity or comorbidity that reported prevalence rates of combinations of two or more diseases.

**Results:**

Our search yielded 3070 potentially eligible articles, of which 19 articles, representing 23 observational studies, turned out to meet all our quality and inclusion criteria after full text review. These studies provided prevalence rates of 165 combinations of two diseases (i.e. disease pairs). Twenty disease pairs, concerning 12 different diseases, were described in at least 3 studies. Depression was found to be the disease that was most commonly clustered, and was paired with 8 different diseases, in the available studies. Hypertension and diabetes mellitus were found to be the second most clustered diseases, both with 6 different diseases. Prevalence rates for each disease combination varied considerably per study, but were highest for the pairs that included hypertension, coronary artery disease, and diabetes mellitus.

**Conclusions:**

Twenty disease pairs were assessed most frequently in patients with multimorbidity. These disease combinations could serve as a first priority setting towards the development of multimorbidity guidelines, starting with the diseases with the highest observed prevalence rates and those with potential interacting treatment plans.

## Introduction

The growing interest in the concept of multimorbidity, which refers to the co-occurrence of multiple (often chronic) diseases or medical conditions within one person[1], is motivated by the rising prevalence of multimorbidity, its negative health consequences, and the challenge to manage multimorbid patients in health care settings, often family medicine practice[2-11]. 

Managing patients with multimorbidity is much more complicated than managing patients with a single condition[10]. Clinical evidence-based guidelines have been developed to provide recommendations for patient management, to define standards of care, and focus efforts to improve quality. However, most clinical guidelines address single diseases, and do not always provide guidance for patients with multimorbidity. Simply combining the current disease oriented guidelines might result in a complex, inconvenient or even conflicting treatment regime, in terms of interactions between drugs and diseases, conflicting management strategies, and polypharmacy[10-12]. To support health care providers in daily practice, guidelines for combinations of diseases are thus warranted, especially for the most prevalent combinations with complex or incompatible regimes. 

Despite the increasing body of research that has been conducted in the field of multimorbidity, there is still no clear, uniform operational definition for multimorbidity, and thus no clear picture of common multimorbidity combinations. Over the years, various methods have been developed and employed to measure multimorbidity. There are indices available that estimate a multimorbidity-score by weighting a range of diseases (e.g. Charlson Comorbidity Index[13] or Cumulative Illness Rating Scale[14]). Other applied multimorbidity measures are the Chronic Disease Score[15], RxRisk Model[16], or the Duke Severity of Illness Checklist[17]. Furthermore, multimorbidity can be assessed by simply counting the number of co-existing diseases within a person, using a predefined list of medical conditions. As disease counts are easy to use, it is presumably the most common approach to define multimorbidity. 

Two recent systematic reviews described the available measures of multimorbidity in more detail and pointed out that the choice of a measure depends on the outcome of interest and the type of data available[18,19]. Overall, these methods are employed to predict health outcomes, for instance, disability, quality of life, health care utilization or mortality. Additionally, these methods are often applied to assess prevalence rates. Prevalence estimates vary widely depending on the study population, setting, data sources, the type of the diseases considered and the number of conditions included in the analysis[18,20-23]. 

Although evidence for the overall prevalence of multimorbidity is accumulating, insight into the prevalence of specific disease combinations (i.e. disease clusters) is limited. A few studies explored disease clusters of multimorbidity by conducting statistical cluster or factor analysis[24-26]. These studies identified several broad clusters of diseases, but it remained unclear which specific combinations of diseases were most frequently occurring, taken into account the variation in prevalence rates. To the best of our knowledge, there are no systematic reviews that have investigated multimorbidity clusters, and therefore, a complete overview is still lacking. 

With this current systematic review we aim to describe the prevalence of disease clusters in older patients with multimorbidity, as found in published studies. In addition, we intend to acquire information that can be supportive in the process of developing multimorbidity guidelines that could assist patient management and improve quality of health care. 

## Methods

### Search strategy

To find eligible studies we consulted the electronic databases MEDLINE/PubMed, Embase and Cochrane Library. A search strategy was developed for each database, using a combination of key words and Medical Subject Headings (MEDLINE) or Emtree terms (EMBASE and Cochrane Library). Since the term multimorbidity does not have an equivalent in the database’s thesaurus, it was only searched as a key word. Until recently, the term comorbidity was used interchangeably with multimorbidity, as it also refers to the co-existence of multiple conditions[1,27]. Hence, both terms and their spelling variations were included in our search algorithm. We combined search terms relating to multimorbidity (e.g. “multimorbid*”, “multiple chronic diseas*”, “multiple illness*”), comorbidity, chronic disease, and the definition or measurement (e.g. “index”, “definition”, “measurement”, “list”, “instrument”). The search strategy was developed iteratively to identify a combination of terms with an acceptable level of sensitivity and specificity. We restricted the search to articles with an available abstract, published in English or Dutch, and those published between January 2000 and September 2012. Before the year 2000, only a few articles had been published on the concept of multimorbidity. We did not restrict the search to a specific study type. To be complete, we also screened reference lists of all included articles. The final search strategy for MEDLINE is given in Appendix S1.

### Study selection

The selection of studies followed several steps. First, different inclusion and exclusion criteria were specified for the selection of studies by title, abstract and full-text (Table **1**). Second, a random sample of fifteen titles was screened by two authors (JS and JK) to control for unclear formulated inclusion and exclusion criteria, before screening all titles of the yielded articles; there was no disagreement or vagueness. Subsequently, one author (JS) screened all titles for relevancy, based on the defined inclusion and exclusion criteria (Table 1). Third, two authors (JK and JS) independently appraised a sample of twenty abstracts. There was no disagreement between the two authors, after which all remaining abstracts were screened for eligibility by one author (JS) and, when necessary, by a second author (JK or JB). Last, full-text articles were independently screened for eligibility by at least two authors (JS screened all the full texts, and JK and JB both screened half of the full texts). To evaluate the full text articles on the inclusion and exclusion criteria, both authors appointed to screen the full text article filled out a self-constructed checklist. Discrepancies and ambiguities were solved by discussion between the two authors and, when necessary, by a third author.

**Table 1:** Inclusion and exclusion criteria of the screening process of the yielded articles.

**Inclusion criteria**		**Exclusion criteria**
Titles	- Included the words ‘multimorbidity’ or ‘comorbidity’ or related words (see step 1 and 2 in Appendix S2)	- No data of disease combinations (or impossible to calculate prevalence rates)
	*Titles not including these words were excluded*	- Age of at least half of the study population was ≤ 55 years
		- Diagnosis of a disease was based on medication prescription (ATC codes) only
Abstracts	- Evidence that multimorbidity/comorbidity was the outcome variable, or the central independent variable	- Study size less than 500 persons^†^
	- Availability of a list of diseases to account for multimorbidity/comorbidity, morbidity indices or measures.	- Study conducted in a hospital setting^‡^
	*Abstracts not meeting these criteria were excluded.*	- Study examined solely two diseases^§^
		- Study was focused on an index-disease with a prevalence < 0.5% in the total population in the Netherlands
Full-texts	- Availability of prevalence rates of specific disease clusters^*^	- Study with a non-empiric research type: ‘letter’, ‘(narrative) review’, ‘editorial’, ‘case-study’, ‘presentation’, ‘commentary’

* or results that allowed the calculation of a prevalence rate: Some studies reported odds ratios instead of prevalence rates. These data were converted into prevalence rates. If not possible, the article was excluded.

^†^ to include studies with results based on solid, robust data

^‡^ our study is more focused on primary care as health professionals in primary care often see patients with multiple health conditions

^§^ we assumed that studies solely focusing on two diseases would provide insufficient disease clusters with applicable prevalence rates

### Assessment of study quality

After titles and abstracts had been screened, all remaining articles had an observational design. Therefore, quality assessment of the articles was based on several items of the Strengthening the Reporting of Observational studies in Epidemiology (STROBE) checklist[28], which we included in our checklist. The items that were required to be described in the articles were (1) the study design; (2) the setting; (3) the study size; (4) eligibility criteria of participants; (5) the type of diseases included to measure comorbidity or multimorbidity; (6) the data collection method; and (7) outcome data related to the prevalence of combinations of diseases. These items, with specific conditions, were also considered as inclusion and exclusion criteria (see also Table 1). In addition, to be retained in our review, only those articles that met our inclusion and exclusion criteria, and thus our specified quality standard, were selected. 

### Data extraction and synthesis

For each included study, the following data were extracted:

1 Study characteristics: First author, year of publication, country, study size, setting, population age;
2 Information relating to the number and types of diseases examined;
3 Information relating to (the prevalence of) the presented disease clusters.

The checklist was employed to gather data about the study characteristics. These data were tabulated and ordered according to the population setting and the presence or absence of a specific index-disease. A mean age was given or calculated, but when impossible the age range was given. Subsequently, all possible diseases, and disease combinations as described in the included studies, were gathered, counted, and tabulated. In addition, the accompanying prevalence rates for each combination were collected and presented. When necessary, odds ratios were converted into prevalence rates. All given prevalence rates concerned the total study sample, and if not, prevalence rates were converted to relate to the total sample.

## Results

### Included studies

**Figure 1:**
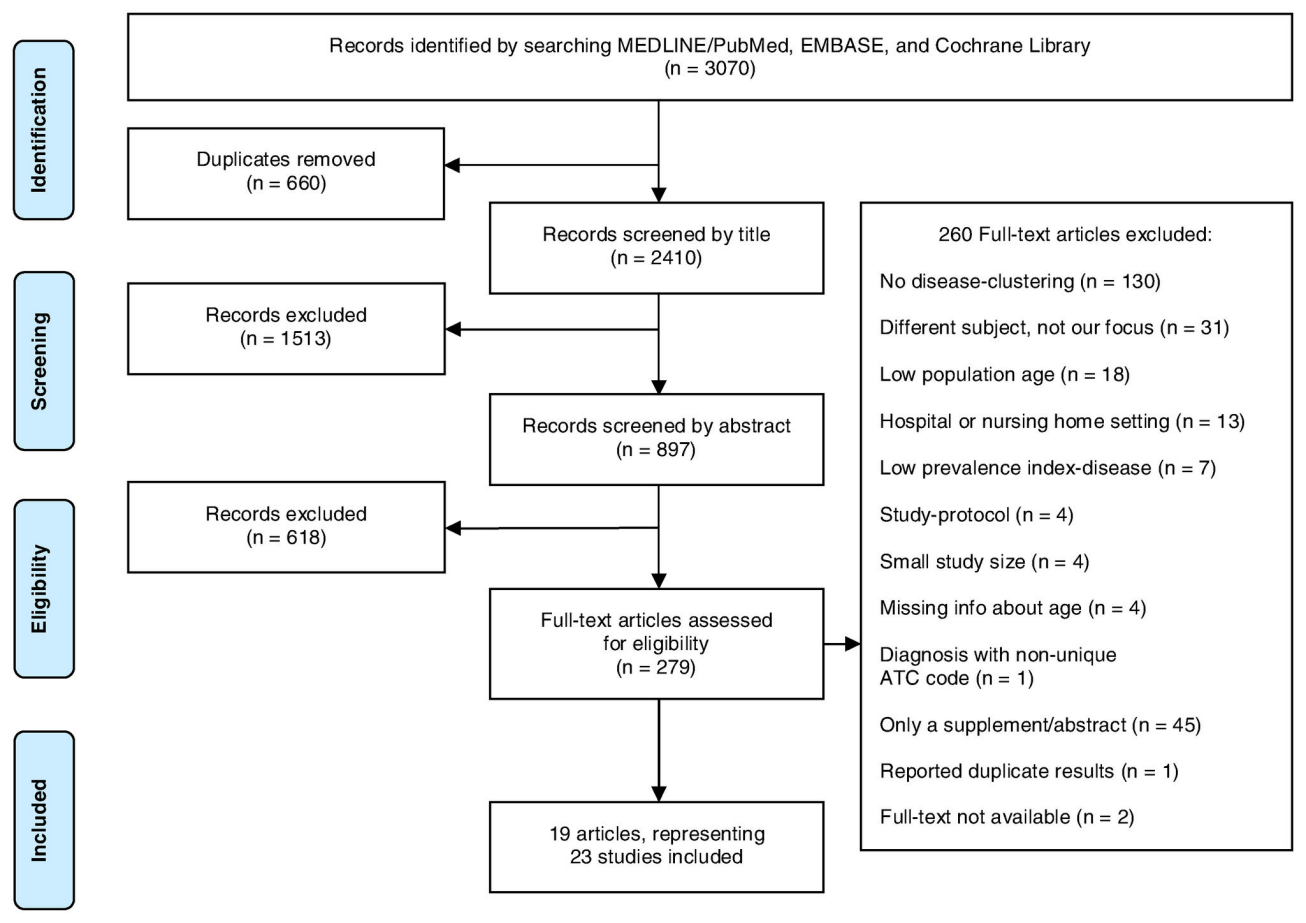
PRISMA Flow chart outlining the study selection process.

In total, 3070 potentially eligible articles were identified, of which 2410 remained after exclusion of duplicates, see Figure **1**. After screening of titles and abstracts, 279 articles remained to be read completely. Of these articles, 212 were excluded because they did not meet our inclusion criteria, as shown in Figure 1. Additionally, 45 articles were found to be an abstract or supplement for a congress and were excluded, 1 article was excluded because of double publication of part of the results of the same research project, and of 2 articles we had no access to the full-text. As a result, 19 articles remained. One of these articles focused on multimorbidity in different settings and described the data of these populations separately. These different settings were regarded as 5 individual studies and therefore, our final sample for analysis represented 23 studies. All 23 studies fulfilled our inclusion criteria and met our quality criteria. 

### Study characteristics

All 23 studies had an observational design and were conducted in either the general population (*n* =13)[23,29-38], primary care (*n* =7)[23,39-43] or ambulatory care setting (*n* =1)[44]. Two studies were based on data of the Veterans Health Administration system (VHA)[6,45] (Table **2**). The population size of the studies varied from 599[23] to over one million[45] individuals. Except for two[44,45], all studies reported clusters of two diseases. In five studies[37,38,42,43,45] patients were only included when diagnosed with a specific disease (i.e. index-disease). In 8 studies[29,30,32-34,36,39,40] prevalence rates were converted to provide comparable prevalence rates of the disease clusters. In one study, odds ratios were converted into prevalence rates[35].

**Table 2:** Characteristics of included studies examining clusters of comorbidity or multimorbidity.

							**Type of diseases/ disease categories examined in the study**										
	**First author (year)**	**Country**	**Setting, (no. of participants used in analyses), Mean age/ percentage**	**Data collection multi/co-morbidity**	**No. of diagnoses examined incl. index-disease**	**Index-disease**	**CVD**	**Diabetes**	**COPD/ asthma**	**Cancer **	**Musculo-skeletal**	**Depression/anxiety**	**Dementia **	**Neurological **	**Eye/ ear **	**Digestive **	**Urinary**
1	Fiest^29^ (2011)	Canada	Gen. pop., (*n*= 15 591), 64 years	Interview with participants	12 (out of 19 diagnoses)	-	yes		yes		yes	yes		yes		yes	
2	Niti^30^ (2007)	Singapore	Gen. pop., (*n*= 2 611), 66 years	Interview with participants	12	-	yes	yes	yes		yes	yes			yes	yes	
3	Marengoni^31^ (2009)	Sweden	Gen. pop, (*n*= 1 099), 85 years	Physician’s examination, hospital records, drug use and clinical examination	11 (out of 15 diagnoses)	-	yes	yes			yes	yes	yes		yes		
4	Kriegsman^32^ (2004)	The Netherlands	Gen. pop., (*n*= 2 497), 69 years	Interview with participants	7	-	yes	yes	yes	yes	yes						
5	Fuchs^33^ (2012)	Germany	Gen. pop., (*n*= 9 155), 56% 55-64 years, 31% 65-74 years, 13% ≥ 75 years	Interview with participants	6	-	yes		yes	yes	yes	yes				yes	
6	Lee P^34^ (2009)	United States	Gen. pop., (*n*= 11 113), 55% 65-75 years, 45% ≥ 76 years	Interview with participants	3 diseases and 2 syndromes	-	yes	yes									yes
7	Fillenbaum^35^ (2000)	United States	Gen. pop., (*n*= 4 034), 73 years	Interview with participants	5	-	yes	yes		yes							
8a	Schram^23^ LASA^*^ (2008)	The Netherlands	Gen. pop., (*n*= 2 463), 55‑94 years	Interview with participants, validated by family physician	5 (out of 10 diagnoses)	-	yes		yes	yes	yes						
8b	Schram^23^ The Rotterdam Study^†^ (2008)	The Netherlands	Gen. pop., (*n*= 3 550), 65‑99 years	Interview with participants, validated by family physician, physical examination	4 (out of 15 diagnoses	-	yes	yes									
8c	Schram^23^ Leiden 85-plus Study^‡^ (2008)	The Netherlands	Gen. pop., (*n*= 599), 85 years	Interview with family physician, electronic medical records	5 (out of 12 diagnoses)	-	yes			yes	yes	yes					
9	Mannino^36^ (2008)	United States	Gen. pop., (*n*= 20 296), 60% ≥ 55 years	Interview with participants, clinical examination	4	-	yes	yes	yes								
10	Wesseling^37^ (2013)^§^	The Netherlands	Gen. pop., (*n*= 979), 56 years	Survey with participants	19 (out of 25 diagnoses)	Osteoarthritis	yes	yes	yes		yes			yes		yes	yes
11	Lyketsos^38^ (2005)	United States	Gen. pop., (*n*= 695), 82 years	Interview with participants	12 (out of 26 diagnoses)	Dementia or Other cognitive impairment	yes	yes			yes		yes	yes		yes	
12	Pfaff^39^ (2009)	Australia	Primary care, (*n*= 20 183) 72 years	Survey with participants	15	-	yes	yes	yes	yes	yes	yes	yes	yes			
13	Schubert^40^ (2006)	United States	Primary care, (*n*= 3 013), 71 years	Electronic medical records	11	-	yes	yes	yes	yes	yes		yes			yes	yes
14	Van Oostrom^41^ (2012)	The Netherlands	Primary care, (*n*= 52 014) 43% 55-64 years 34% 65-74 years 23% ≥ 75 years	Electronic medical records	10 (out of 29 diagnoses)	-	yes	yes	yes	yes	yes	yes					
8d	Schram^23^ CMR Nijmegen^||^ (2008)	The Netherlands	Primary care, (*n*= 2 895) 100% ≥ 55 years	Electronic medical records	6 (out of a total of 68 diagnoses)	-	yes^¶^	yes			yes				yes		
8e	Schram^23^ RNGP^**^ (2008)	The Netherlands	Primary care, (*n*= 5 610) 100% ≥ 55 years	Electronic medical records	6 (out of a total of 83 diagnoses)	-	yes	yes		yes	yes						
15	Noël^42^ (2004)	United States	Primary care, (*n*= 1 801) 77% ≥ 65 years	Interview with participants	11	Major depression or dysthymia	yes	yes	yes	yes	yes	yes		yes		yes	yes
16	Struijs^43^ (2006)	The Netherlands	Primary care, (*n*= 7 499) 65 years	Electronic medical records	11	Diabetes mellitus	yes	yes	yes	yes	yes	yes		yes	yes		yes
17	Findley^45^ (2011)	United States	VHA clinical services users (veterans), (*n*= 1 383 950) 90% ≥ 50 years	VHA electronic medical records and Medicare claims data	4	Diabetes mellitus, heart disease, hypertension	yes	yes				yes					
18	Lee T^6^ (2007)	United States	VHA clinical services users (veterans), (*n*= 741 847) 100% 55‑64 years	VHA electronic medical records	6 (out of 11 diagnoses)	-	yes	yes	yes	yes	yes						
19	Van den Bussche^44^ (2011)	Germany	Ambulatory care, (*n*= 123 224) 74 years	Claims data	19 (out of 46 diagnoses)	-	yes	yes	yes	yes	yes	yes			yes	yes	

Gen. pop.: General population; CVD: cardiovascular diseases; VHA: Veterans Health Administration system

* Schram et al. analyzed data from seven registries, these are presented separately. This is data from a population-based registry, LASA.

^†^ Schram et al. analyzed data from seven registries, these are presented separately. This is data from a population-based registry, The Rotterdam Study.

^‡^ Schram et al. analyzed data from seven registries, these are presented separately. This is data from a population-based registry, Leiden 85-plus Study.

^§^ During the search, this was still a provisional publication

^||^ Schram et al. analyzed data from seven registries, these are presented separately. This is data from a primary care registry, CMR Nijmegen.

^¶^ hypertension only

** Schram et al. analyzed data from seven registries, these are presented separately. This is data from a primary care registry, RNGP.

### Type of diseases

**Figure 2:**
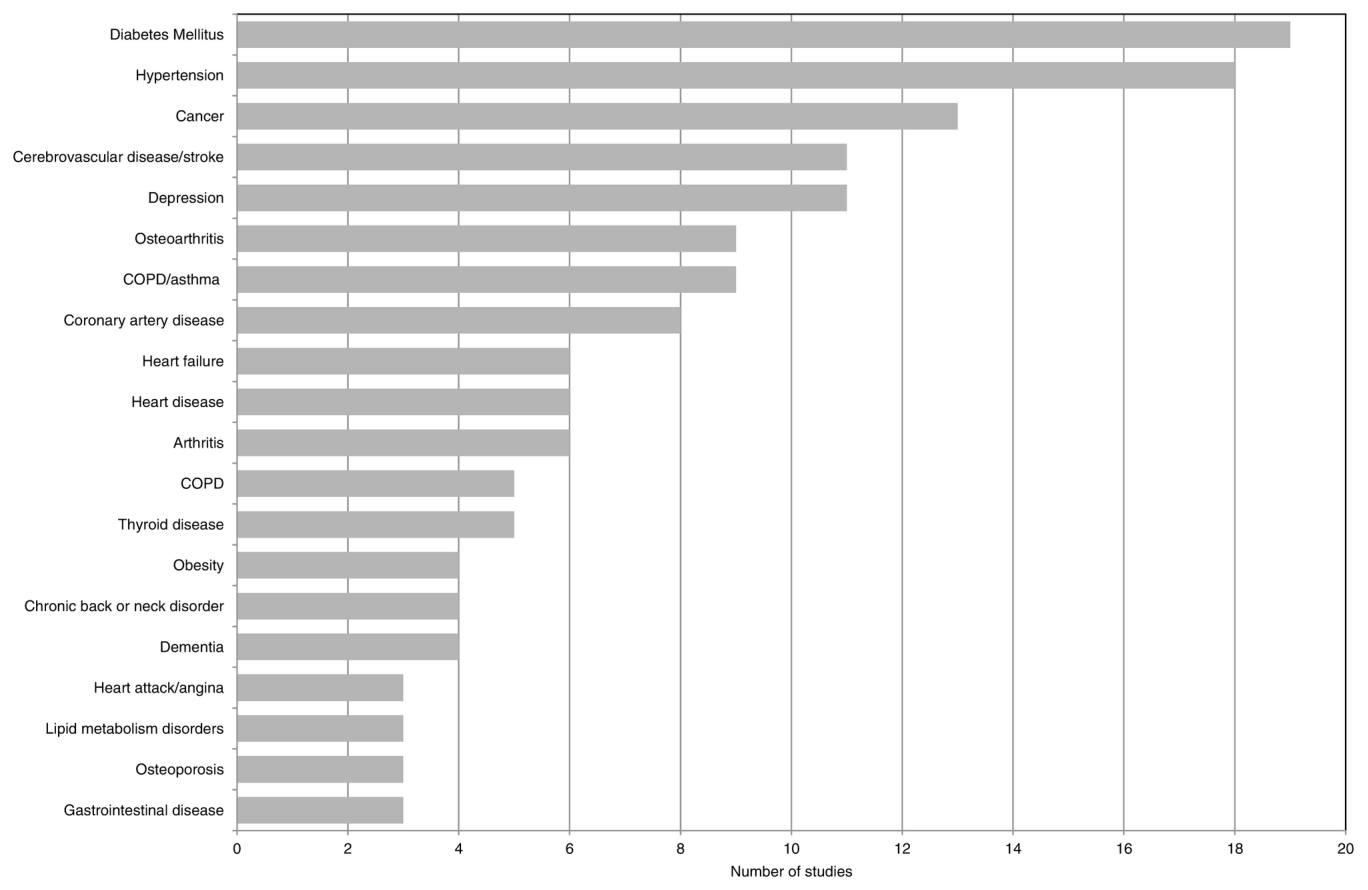
Type of diseases examined in the included studies (top 20).

Sixty-three different diseases were found, of which some were defined rather broadly (e.g. heart disease, gastrointestinal disease), while others were described in more detail (e.g. cataract, atrial fibrillation). Diabetes mellitus was the most frequently measured disease (described in 19 out of 23 studies). Other commonly assessed diseases were hypertension, cancer, stroke, and depression (Figure **2**). Besides the 63 diseases, 165 combinations of two diseases (i.e. disease pairs) and 50 combinations of three diseases (i.e. disease triplets) were reported in the studies. Of the disease pairs, 20 were described rather frequently (≥ 3 studies), see Table **3**. The disease triplets could not be replicated in any of the other studies and were therefore not further analyzed. 

**Table 3:** Prevalence of clusters of two diseases.

**Disease**	**Clustered with**	Prevalence per study (%; %; %), data gathered by an interview/survey*	Prevalence per study (%; %; %), data collected by patients’ EMRs*	No. of study*
Depression	Hypertension	**1.2**; 3.9; **7.6**; **12.9**		**1**, 12, **2**, **8c**
	Arthritis	**1.7**; **2.8**; 4.9		**1**, **2**, 12
	Diabetes Mellitus	1.7; **2.8**	1.4	12, **2**, 14
	COPD/Asthma	**0.9**; 1.8		**2**, 12
	Stroke	**0.2**; **0.9**; 1.0	0.8; **1.1**	**1**, **2**, 12, 14, **3**
	Cancer	1.1	0.9	12, 14
	Heart failure	0.7; **0.8**	0.7	12, **2**, 14
	Heart disease		0.6	**1**
Hypertension	Osteoarthritis	**18.7**; **20.1**	*3.2*; 4.1; 9.1	**8c**, **8a**, *18*, 8e, 8d
	Coronary artery disease	**9.8**; **14.9**	**7.6**	**7, 8a, 3**
	Diabetes Mellitus	**12.0**; **14.0**	**2.5**; 6.2; *6.4*; 7.4	**8b, 7, 3**, 8e, *18*, 8d
	Cancer	**5.5**; **10.6**	*1.0*; 3.4	**7, 8c**, *18*, 8e
	Depression	**1.2**; 3.9; **7.6**; **12.9**		**1**, 12, **2**, **8c**
	Dementia		2.9; **5.5**	13, **3**
Diabetes Mellitus	Hypertension	**12.0**; **14.0**	**2.5**; 6.2; *6.4*; 7.4	**8b, 7, 3**, 8e, *18*, 8d
	Coronary artery disease	**4.1**; **4.5**	3.6	**7, 6**, 14
	Stroke	**0.6**; **2.9**	1.9	**4**, **7**, 14
	Depression	1.7; **2.8**	1.4	12, **2**, 14
	Heart failure	**1.8**	**1.8**; 2.2	**6,** 3,14
	Cancer	**0.8**; **2.2**	1.9	**4, 7**, 14
Cancer	Hypertension	**5.5**; **10.6**	*1.0*; 3.4	**7, 8c**, *18*, 8e
	Diabetes Mellitus	**0.8**; **2.2**	1.9	**4, 7**, 14
	Depression	1.1	0.9	12, 14
	Stroke	**0.5**; **0.9**	0.9	**4, 7**, 14
Stroke	Diabetes Mellitus	**0.6**; **2.9**	1.9	**4, 7**, 14
	Dementia		0.4; **2.7**	13,3
	Depression	**0.2**; **0.9**; 1.0	0.8; **1.1**	**1**, **2**, 12, 14, **3**
	Cancer	**0.5**; **0.9**	0.9	**4, 7**, 14
Coronary artery disease	Hypertension	**9.8**; **14.9**	**7.6**	**7, 8a, 3**
	Heart failure	**2.8**	2.8; **5.6**	**6**, 14, **3**
	Diabetes Mellitus	**4.1**; **4.5**	3.6	**7, 6**, 14
Heart failure	Coronary artery disease	**2.8**	2.8; **5.6**	**6**, 14, **3**
	Diabetes Mellitus	**1.8**	**1.8**; 2.2	**6,** 3,14
	Depression	0.7; **0.8**	0.7	12, **2**, 14
Dementia	Hypertension		2.9; **5.5**	13, **3**
	Stroke		0.4; **2.7**	13, **3**
Osteoarthritis	Hypertension	**18.7**; **20.1**	*3.2*; 4.1; 9.1	**8c, 8a**, *18*, 8e, 8d
Arthritis	Depression	**1.7**; **2.8**; 4.9		**1, 2**, 12
COPD/Asthma	Depression	**0.9**; 1.8		**2**, 12
Heart disease	Depression	**0.6**		**1**

Prevalence of disease clusters found in at least three studies

EMR: Electronic medical record

* Not bold: studies conducted in a primary care setting, **bold**: studies conducted in the general population, *italic*: study based on VHA (Veterans Health Administration system) data.

The rank in frequency of diseases examined in the included studies depended on the definition of the diseases. As displayed in Figure 2, various diseases of the circulatory tract were examined frequently (6 diseases in the top 20). However, the definition of these diseases differed in level of detail. If heart failure, coronary artery disease and heart attack/angina were defined as heart disease (this broad definition could comprise the separate diseases), heart disease was examined in 17 studies instead of in 6 (in some studies coronary artery disease and heart failure were both examined), making it the third most commonly assessed disease. This also applied the category COPD/asthma and the separate diseases asthma and COPD. If the specific diseases were grouped into the broad combined category, then COPD/asthma was investigated in 14 studies, instead of in 9 studies. 

### Disease clusters

The most frequently assessed combinations concerned 12 different diseases (Table 3). Regarding these diseases, several clusters were identified. Of the assessed diseases, depression was most frequently clustered, and was paired with 8 other diseases. Additionally, hypertension and diabetes mellitus were also found to be commonly clustered in the available studies (with 6 different diseases). Although depression was the disease most frequently assessed in pairs, the highest prevalence rates were found for disease pairs including hypertension, highest for its combination with osteoarthritis (20%). The top ten disease combinations with the highest prevalence rates all included the diseases hypertension, coronary artery disease, and diabetes mellitus. In the studies that focused on a specific index-disease, mainly studies concerning depression, even higher prevalence rates were identified; 57% of the patients with a major depression were also diagnosed with hypertension (see Table **4**). 

**Table 4:** Prevalence of clusters of two diseases, including an index-disease.

**Index-disease**	**Clustered with**	Prevalence per study (%; %; %), data gathered by an interview/survey*	Prevalence per study (%; %; %), data collected by patients’ EMRs*	No. of study*
Depression	Hypertension	57.9		15
	Arthritis	55.6		15
	Diabetes Mellitus	23.2		15
	COPD/Asthma	23.3		15
	Cancer	10.9		15
	Heart disease	27.6		15
Hypertension	Depression		*16.7*	*17*
Diabetes Mellitus	Stroke		2.9	16
	Depression		3.9; *17.6*	16, *17*
	Cancer		2.7	16
Dementia	Hypertension	**37.1**		**11**
	Stroke	**16.4**		**11**
Osteoarthritis	Hypertension	**19.8**		**10**
Heart disease	Depression		*16.6*	*17*

Prevalence of disease clusters found in at least three studies

EMR: Electronic medical record

* Not bold: studies conducted in a primary care setting, **bold**: studies conducted in the general population, *italics*: study based on VHA (Veterans Health Administration system) data.

Per study, varying prevalence rates for each disease combination were found. Especially for depression with hypertension (from 1.2% to 12.9%), and for cancer with hypertension (from 1.0% to 10.6%). Further, the highest prevalence values were often found in studies in which the morbidity data were collected via interviews or surveys. These studies almost always concerned the general population. Nearly all studies that applied electronic medical records (EMRs) to collect morbidity data were executed in a primary care setting. 

## Discussion

While multimorbidity in older people seems to be the rule rather than the exception, evidence on the prevalence of specific disease clusters in patients with multimorbidity is limited. In this systematic review 19 articles were included, representing 23 studies, that described 63 diseases and 165 disease pairs. Twenty disease pairs, comprising 12 different diseases, were examined rather frequently. Of the assessed diseases, depression was the disease most frequently clustered, and was paired with 8 different diseases. Hypertension and diabetes mellitus were found to be the second most commonly clustered diseases, and were combined with 6 different diseases. The combinations with the highest prevalence rates included hypertension, coronary artery disease and diabetes mellitus. 

The prevalence estimates of disease clusters differed widely among studies, a result that is in line with findings reported in other reviews[20,46]. We will discuss two main possible explanations. First, differences in the population under study may affect the prevalence of multimorbidity and related disease clusters, like age, income, or ethnicity[47-52]. Multimorbidity is strongly associated with age[47-50]. Although we focused on older adults, the population’s mean age still varied considerably (from 56 years to 85 years). Further, multimorbidity seems more common among people living in socioeconomically deprived areas or people with a low income[47,49,50]. Second, variation in prevalence rates might be due to the applied definition of the diseases, the applied data collection method and the study setting[18-21,53,54]. In our review, some diseases were defined very broadly (e.g. cancer, heart disease) while other diseases were defined in more detail (e.g. osteoarthritis, atrial fibrillation). Studies executed in a primary care setting often applied medical records with information on a detailed level, yet they applied different classification codes with different definitions or based on different diagnostic methods (e.g. depression). In contrast, studies applied in the general population often used surveys or interviews, all inquiring about diseases differently. Other diseases, like obesity, are not always considered as a disease and therefore not included. As a consequence, few disease combinations and accompanying prevalence rates were identical. 

With our current results we have identified combinations of diseases that are likely to co-occur and thus, a suitable treatment plan needs to be developed. Existing clinical practice guidelines, however, do not often address multimorbidity, and following all guidelines for all individual diseases may lead to a considerable treatment burden and to contradictory drug and self-care regimes[10,11,55]. Indeed, Boyd et al.[10] reported that several potential medication interactions were found for a pattern that consisted of the diseases hypertension, diabetes mellitus, osteoarthritis, osteoporosis, and COPD. Contradicting life-style recommendations were found for osteoporosis and diabetes mellitus.. As it is reasonable that our identified disease pairs are highly common in (elderly) adults, it would be useful if guidelines address potential drug interactions and contradicting treatment recommendations (drug-disease interactions, and disease-disease interactions) for these disease pairs. 

This systematic review has some limitations. We used the term multimorbidity in our search process. This term is not well indexed in literature databases, and we might have missed some studies. To compensate for this constraint, we combined an extended list of text words referring to the term multimorbidity and we included the term comorbidity (with its possible spelling variations) to our search strategy. Next, we developed a scoring method based on several items of the STROBE checklist[28], and added these items to our strict inclusion and exclusion criteria, in order to obtain a minimal quality standard of all included studies. As a result, we could not differentiate further between levels of quality. Last, with this type of study we were restricted to merely describe the most *frequently explored* disease pairs in patients with multimorbidity, and not necessarily the *most occurring* disease pairs. Yet, the 12 identified diseases do represent highly prevalent diseases internationally[56,57], and the accompanying combinations of these diseases are also likely to be highly prevalent. 

Reflecting on our findings and limitations, more effort should be made to establish a multimorbidity disease list with uniformly defined diseases. Only by doing so, heterogeneity between study results can be diminished, and information about the prevalence and burden of multimorbidity will be more genuine and comparable. It seems also important to have a better understanding of specific treatment conflicts concerning certain disease clusters, and not merely by scrutinizing the existing guidelines, but by actually assessing daily practice according to guideline recommendations. In this regard, it seems practical to start with the most frequently occurring diseases. Furthermore, it is still valuable to gain more insight into (the prevalence of) specific co-occurring disease clusters, especially of clusters of three, and four diseases, as a large proportion of the elderly population is diagnosed with more than two chronic conditions[50]. For the development of a multimorbidity guideline, however, it might be easier to take into account rather small disease clusters instead of broad, comprehensive disease clusters[25,26]. 

## Conclusion

Management of care for (older) patients with multimorbidity can be challenging, or even burdensome. To be more concrete, health professionals need to strike a balance between the various disease-specific guidelines before one can develop an appropriate treatment plan with feasible recommendations and advices, taking the patient’s personal abilities into account. The disease clusters that we have distinguished, could serve as a first priority setting towards the development of multimorbidity guidelines. A likely option is to start with the most frequently occurring disease combinations, as regards the evaluation of potential treatment conflicts, the adjustment of existing clinical guidelines, or even the development of new guidelines. 

## Supporting Information

Appendix S1 **Electronic literature search of PubMed/MEDLINE, September 2012.**
(DOC)

Checklist S1 **PRISMA checklist.**
(DOC)
