# Supplementary material for: The Prevalence of Disease Clusters in Older Adults with Multiple Chronic Diseases – A Systematic Literature Review
Source: PLoS One. 2013 Nov 11;8(11):e79641. doi: 10.1371/journal.pone.0079641 (PMC3823581; doi:10.1371/journal.pone.0079641)
Supplement: Appendix S1 — Electronic literature search of PubMed/MEDLINE, September 2012. (DOC) [file pone.0079641.s001.doc]

| **Step** | **Search terms** | **Studies found** |
| --- | --- | --- |
| 1 | multimorbid*[tiab] OR multi-morbid*[tiab] OR multiple morbidit*[tiab] OR multiple diseas*[tiab] OR multiple illness*[tiab] OR multiple diagnos*[tiab] OR multiple chronic diseas*[tiab] OR multiple chronic illness*[tiab] OR multiple chronic diagnos*[tiab] OR multiple chronic condition*[tiab] | 2 878 |
| 2 | comorbidity[Mesh] OR comorbid*[ti] OR co‑morbid*[ti] | 58 970 |
| 3 | “chronic disease”[Mesh] | 206 793 |
| 4 | #2 AND #3 | 2 969 |
| 5 | measure[tiab] OR measured[tiab] OR measurement[tiab] OR measurements[tiab] OR measures[tiab] OR index[tiab] OR indexes[tiab] OR indexed[tiab] OR indices[tiab] OR list[tiab] OR listed[tiab] OR lists[tiab] OR classification[tiab] OR classifications[tiab] OR classified[tiab] OR classifies[tiab] OR classify[tiab] OR classifying[tiab] OR instrument[tiab] OR instruments[tiab] OR definition[tiab] OR define[tiab] OR defined[tiab] OR scale[tiab] | 3 342 139 |
| 6 | #1 AND #5 | 921 |
| 7 | #4 AND #5 | 1 230 |
| 8 | #6 OR #7 | 2 071 |
| 9 | articles with an abstract available | 2 064 |
| 10 | limit #9 to English or Dutch language | 1 835 |
| 11 | limit #10 to publications from the year 2000 to current | 1 602 |
